# Achieving Maximum Chirality and Enhancing Third-Harmonic Generation via Quasi-Bound States in the Continuum in Nonlinear Metasurfaces

**DOI:** 10.3390/nano16070388

**Published:** 2026-03-24

**Authors:** Du Li, Yuchang Liu, Kun Liang, Li Yu

**Affiliations:** State Key Laboratory of Information Photonics and Optical Communications, School of Physical Science and Technology, Beijing University of Posts and Telecommunications, Beijing 100876, China; ld1997@bupt.edu.cn (D.L.); yuchangliu@bupt.edu.cn (Y.L.); yuliyuli@bupt.edu.cn (L.Y.)

**Keywords:** bound states in the continuum, nonlinear, chirality

## Abstract

Chiral bound states in the continuum (BIC) metasurfaces have emerged as a promising platform for enhancing light–matter interactions, which have potential applications in advanced photonic and quantum information devices. However, simultaneously achieving near-perfect circular dichroism and highly efficient nonlinear conversion with highly symmetric structures in metasurfaces remains an open challenge. In this work, we design a $C_{4}$-symmetric chiral metasurface composed of eight elliptical silicon nanorods on a ${\mathrm{SiO}}_{2}$ substrate, where monocrystalline silicon is used as the nonlinear optical material. By combining simulations and nonlinear time-domain coupled-mode theory (TCMT), we discovered that both the optimal chirality and the nonlinear conversion efficiency can be attained simultaneously due to the critical coupling between the metasurface mode and the quasi-BIC mode. Meanwhile, a near-perfect circular dichroism (CD = 0.99) and a high nonlinear conversion efficiency of $7\times 10^{-5}$ under a radiation intensity of $5\;{\mathrm{kW}/\mathrm{cm}}^{2}$ are numerically achieved due to the robustness of bound states in the continuum. This work offers a promising route toward high-performance chiral nonlinear photonic components, which is of great importance for the development of ultra-compact optical devices such as circular polarization detectors, chiral sensors, and nonlinear photonic chips for integrated optical and quantum information systems. Our research not only contributes to the fundamental understanding of chiral metasurfaces but also provides a practical approach for achieving high-efficiency nonlinear optical devices.

## 1. Introduction

The manipulation of light–matter interactions at the nanoscale has attracted significant attention due to its potential applications in advanced photonic and quantum information devices [1,2,3,4]. Among various approaches, chiral metasurfaces [5] have emerged as a promising platform to control chiroptical responses, offering unique opportunities to enhance circular dichroism (CD) and nonlinear optical processes [6,7]. Recent studies have demonstrated that chiral metasurfaces can achieve remarkable CD enhancements [8,9] and efficient nonlinear optical effects, such as second harmonic generation (SHG) [10,11] and third harmonic generation (THG) [12]. These advances are crucial for applications in optical sensing [13,14], imaging [15,16], and information processing [17].

In particular, the integration of bound states in the continuum (BICs) with chiral metasurfaces has opened new avenues for achieving high-Q-resonances and strong light–matter interactions [18,19]. BICs are unique states that remain confined within a continuous spectrum, exhibiting infinite radiation Q-factors in ideal conditions [20]. When combined with chiral structures, BICs can significantly enhance the local electromagnetic fields [21], leading to pronounced nonlinear optical responses. Chiral quasi-BIC modes originate from the symmetry breaking of ideal BICs with infinite *Q*-factors, where ultra-high *Q*-factors and strong local electromagnetic field confinement enable significant enhancement of light–matter interactions. For dielectric metasurfaces, the efficiency of third-harmonic generation is determined by two core factors, namely, the intensity of local field enhancement and the spatial overlap between the enhanced field and the nonlinear material, both of which are inherently optimized by quasi-BICs. For example, recent work has shown that chiral BIC metasurfaces can achieve high CD values and efficient SHG through a careful design of the geometry of the metasurface and the material properties [22,23,24,25]. Moreover, the exploration of THG in chiral metasurfaces has revealed new opportunities to improve chiroptical effects [26,27,28]. THG can provide additional degrees of freedom to control light-matter interactions, enabling more efficient and versatile nonlinear optical devices [29,30,31]. Meanwhile, studies have shown that chiral metasurfaces can achieve significant THG enhancements [32]. These results highlight the potential of chiral BIC metasurfaces for the realization of high-efficiency nonlinear optical processes.

However, achieving near-perfect chirality [33,34] and highly efficient nonlinear conversion [35,36,37,38,39,40] with highly symmetric structures in the metasurface remains an open challenge. Some previous work achieved high CDs relying on extrinsic chirality effects while their Q-factors are still small [41,42,43]. Thus, quasi-BIC modes were introduced to combine nonlinearity with chiral metasurface in order to simultaneously enhance the chirality and nonlinearity of the structure and unlock third-harmonic conversion by engineering critical coupling between modes in the metasurface without altering $C_{4}$ symmetry.

In this work, we designed a nonlinear chiral metasurface based on BIC to achieve near-perfect chirality in the third-harmonic band and highly efficient nonlinear conversion while avoiding the influence of pseudochirality. First, we employed nonlinear coupled-mode theory to establish a theoretical model that elucidates the nonlinear and chiral properties of the metasurface. Secondly, we designed a periodic metasurface structure composed of eight silicon rods with $C_{4}$ symmetry. Numerical simulations revealed a symmetry-protected BIC mode in the metasurface, with a resonance wavelength of 1800nm and a *Q* factor of up to $1.8\times 10^{5}$. By breaking the in-plane inversion symmetry of the structure, we successfully generated chiral quasi-BICs. We found that the presence of critical coupling effects [44] within the structure, particularly loss matching between modes in the metasurface, enables effective modulation and amplification of the structural chirality and nonlinear conversion efficiency. Third, by fine-tuning the geometric parameters of the metasurface, such as the structure height and rotation angle, we optimized its nonlinear conversion efficiency and chiral performance. We numerically achieved near-perfect circular dichroism and a nonlinear conversion efficiency of $7\times 10^{-5}$ under a radiation intensity of $5\;{\mathrm{kW}/\mathrm{cm}}^{2}$ in third-harmonic generation. These results were in excellent agreement with theoretical analysis. Our research is positioned as a continuation and extension of the research on BIC-enhanced light–matter interaction, a field significantly advanced by Kivshar and colleagues [22,39]. By highlighting the overlaps with these previous chiral quasi-BIC mechanisms, we demonstrate how our $C_{4}$-symmetric structure further optimizes the interplay between chirality and third-harmonic generation (THG) under a unified framework. Our research not only provides new insights into the application of metasurfaces in nonlinear and chiral optics but also offers a theoretical foundation for further explorations and optimization of metasurface performance.

## 2. Metasurface Design and Methods

### 2.1. Structure Parameter

**Figure 1:**
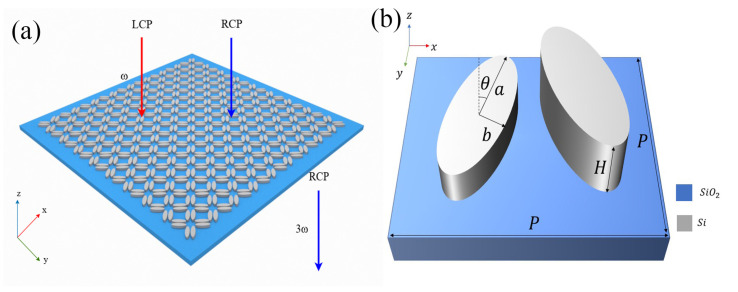
(**a**) Schematic of the nonlinear maximum chirality metasurface based on $C_{4}$-symmetric elliptical Si nanorods; (**b**) Unit cell structure and geometric parameters (a=321 nm, b=102 nm, H=290 nm, P=1400 nm). This $C_{4}$-symmetric structure supports symmetry-protected BIC modes and induces chiral quasi-BICs via tuning structural parameters (δH, δθ).

As in Figure 1, the metasurface is designed with a periodic array of elliptical Si nanorods arranged in a unit cell that exhibits $C_{4}$ symmetry. The periodicity of the metasurface is *P* = 1400 nm, and each unit cell comprises eight elliptical nanorods. The elliptical nanorods have a long semi-axis of *a* = 321 nm, a short semi-axis of *b* = 102 nm, and a height of *H* = 290 nm. The metasurface is placed on a dielectric substrate with a refractive index of $n_{\mathrm{substrate}}=1.465$, and the refractive index of the Si bar is 3.45. The finite element method is used for numerical calculation in this study. The radiation intensity is given by $P_{in}=E_{0}/P^{2}$ = 5 kW/cm^2^. The periodic boundary conditions are set in the *x* and *y* directions of the unit cell, and the perfect matching layers are used in the *z* direction.

These structural parameters are chosen based on the optimization of the metasurface’s ability to support bound states in the continuum and to enhance the third-order nonlinear chiral response. The nonlinear polarization at 3ω can be obtained by utilizing the linear electric E(ω) field at the pump fundamental frequency (FF) [45]

(1) $$P\left(3\omega \right)=3\varepsilon_{0}\chi^{\left(3\right)}\left(E\;\cdot \;E\right)E\left(\omega \right),$$

where $\varepsilon_{0}$ denotes the permittivity in the vacuum, and $\chi^{\left(3\right)}=2.45\times 10^{-19}\;m^{2}V^{-2}$ is the third-order nonlinear coefficient of the Si material which provides nonlinearity in our structure.

This work is a theoretical and numerical simulation study focused on the design and performance optimization of chiral nonlinear metasurfaces. The monocrystalline silicon selected as the core nonlinear material is a mature experimental material with well-characterized physical parameters. Its third-order nonlinear susceptibility $\chi^{\left(3\right)}=2.45\times 10^{-19}\;m^{2}/V^{2}$ and refractive index n=3.45 at 1800 nm are key parameters incorporated into both finite element method (FEM) numerical simulations and nonlinear time-domain coupled-mode theory (TCMT) models. These parameters directly determine the electromagnetic field distribution in FEM simulations as well as the nonlinear polarization term P(3ω) in the TCMT model (Equation (1)).

The proposed metasurface is designed with experimental feasibility in mind, leveraging standard nanofabrication workflows. While our simulations explore fine structural perturbations at $0.5^{\circ }$ angular intervals, such precision is well within the technical limits of contemporary electron beam lithography (EBL). Specifically, modern EBL systems provide the sub-10 nm positioning accuracy and high overlay precision required to define such subtle asymmetries [46]. Furthermore, the inherent robustness of the symmetry-protected BIC ensures that the high-Q-resonance remains experimentally observable even in the presence of minor fabrication tolerances, such as slight corner rounding or etching deviations, as evidenced by similar successful implementations in recent quasi-BIC literature [22,47].

### 2.2. Nonlinear Coupled Mode Theory

To analyze the third-order nonlinear chiral response of the proposed metasurface, we employ the nonlinear TCMT. This theoretical framework allows us to describe the interaction between the incident light and the metasurface’s resonant modes, particularly focusing on the bound states in the continuum supported by the elliptical Si nanorods.

The nonlinear TCMT [48] is based on the interaction of multiple resonant modes within the metasurface, where the evolution of the mode amplitudes is governed by a set of coupled differential equations. For a metasurface supporting multiple resonant modes, the coupled-mode equations can be written as:

(2) $$-i\omega P_{0}=-i\left(\omega_{0}-i\gamma_{0}\right)P_{0}+\langle M|a^{\left(\omega \right)}\rangle$$

where $P_{0}$ refers to the modal resonance amplitude, $\omega_{0}$ is resonance frequency, $\gamma_{0}$ is damping factor of the oscillator, M stand for the operator associated with the coupling coefficient which quantifies the strength of interaction between different modes or between light and the structure and $a^{\left(\omega \right)}$ is the incident light at fundamental frequency. Since the relation between coupling factor and output light $b^{\left(3\omega \right)}$ is $b^{\left(3\omega \right)}\rangle =|N^{\left(3\omega \right)}\rangle P_{0}^{3}$, we can connect the incident light and transmitted light through the structural amplitude.

In the proposed TCMT framework, the back-conversion from 3ω to ω is neglected. This is justified by the fact that under the relatively low pump intensity (5 kW/cm^2^) used in our study, the pump depletion is minimal, and the nonlinear interaction remains in the weak-coupling regime. Furthermore, while alternative CMT versions, such as the doubly resonant model developed by the MIT group [40], provide a more general framework for high-power regimes, we adopted the model from Ref. [48] instead of those from Refs. [45,49,50] because it is more physically intuitive for single-resonance-driven $C_{4}$ symmetric systems and specifically accounts for the phase-matching-like conditions via complex coupling coefficients. In the context of our metasurface, the elliptical Si nanorods support BICs, which are characterized by high-quality factors and strong field confinement. These BICs play a crucial role in enhancing the nonlinear interactions. The coupling coefficients $\kappa_{mn}$ are determined by the spatial overlap between the resonant modes and the incident light, while the nonlinear coefficient $\gamma_{m}$ is related to the third-order susceptibility of silicon and the mode profile.Therefore, the nonlinear scattering matrix can be written as:

(3) $${\hat{S}}^{\left(3\omega \right)}=\frac{|N^{\left(3\omega \right)}\rangle \langle M^{3}|}{i{\left(\omega -\omega_{0}+i\gamma_{0}\right)}^{3}}$$

The third-harmonic vector $N^{\left(3\omega \right)}$ is given by

(4) $$\begin{array}{cc} N^{\left(3\omega \right)}={\left(\frac{i}{c}\right)}^{1/2}{\left(3\omega \right)}^{2} & \sum_{ijkl}\chi_{ijkl}^{\left(3\right)}\int dr^{\prime }E_{0,j}\left(r^{\prime }\right)E_{0,k}\left(r^{\prime }\right)E_{0,l}\left(r^{\prime }\right)u_{\alpha ,i}\left(r^{\prime };3\omega \right) \end{array}$$

where $\chi_{i(j3kl)}$ represents the susceptibility tensor of silicon (Si) material, and $E_{0,j(kl)}\left(r^{\prime }\right)$ denotes the electric field intensity in the *x*, *y*, and *z* directions. The quantity $N_{3\omega }$ is derived from the 3ω mode profile $u_{\alpha ,i}\left(r^{\prime };3\omega \right)$, which is obtained from finite element method (FEM) eigenmode simulations. This profile directly enhances the spatial overlap of $E_{0}\left(r^{\prime }\right)$ with Si nanorods, thereby increasing both $N_{3\omega }$ and the intensity of third-harmonic generation (THG). Equation (4) is used to explicitly describe $u_{\alpha ,i}\left(r^{\prime };3\omega \right)$ as the 3ω mode profile, underscoring the mode’s influence. As in Equation (2), by solving the coupled-mode equations numerically, we can predict the chiral nonlinear response of the metasurface and optimize the structural parameters to maximize the chirality. To maximize the structural chirality, it is critical to enhance the input signal $\langle M|a^{\left(\omega \right)}\rangle$, as it directly determines the magnitude of $P_{0}$. Optimizing the geometric layout of the metasurface elements (e.g., rod height and orientation) improves the local field distribution. Tailoring the material properties to enable higher-order asymmetric resonances further boosts the coupling efficiency at specific chiroptical frequencies. These approaches lead to an enhanced overall chiral signal, as $P_{0}$ scales proportionally with $\langle M|a^{\left(\omega \right)}\rangle$. This theoretical framework provides a comprehensive understanding of the underlying mechanisms and guides the design of the metasurface for enhanced chiral nonlinear interactions.

To ensure the credibility of the theoretical model, all CMT parameters are directly extracted from the FEM-simulated linear scattering spectra of the metasurface. Specifically, the resonance frequency $\omega_{0}$ and the radiative decay rate $\gamma_{rad}$ are determined by fitting the Fano resonance profiles. The excellent agreement between the FEM and CMT results from our simulation is achieved without using any arbitrary adjustable parameters, as the coupling coefficients are derived from the actual near-field overlap integrals, reflecting the intrinsic light–matter interaction within the resonators.

(5) $$CD^{\left(3\omega \right)}=\frac{|N_{R^{\prime }}^{\left(3\omega \right)}{|}^{2}|M_{R}{|}^{6}-|N_{L^{\prime }}^{\left(3\omega \right)}{|}^{2}{\left|M_{L}\right|}^{6}}{|N_{R^{\prime }}^{\left(3\omega \right)}{|}^{2}|M_{R}{|}^{6}+|N_{L^{\prime }}^{\left(3\omega \right)}{|}^{2}{\left|M_{L}\right|}^{6}}$$

where $M_{R(L)}$ are coupling coefficients of the RCP- (LCP-) polarized pump with the resonant mode, *R*($R^{\prime }$) and *L* ($L^{\prime }$) stand for right-handed and left-handed incident light from above (below) individually, $\kappa_{R(L)}\left(3\omega \right)$ describe the nonlinear interaction and outcoupling efficiency at the TH wavelength. In the linear regime, the coupling strength between each polarization state and the system’s eigenmodes (e.g., waveguide modes, surface plasmon polariton modes) is determined by the matching degree of their polarization properties. This mismatch leads to distinct linear coupling coefficients, which can be quantified and modeled using coupling mode theory (CMT) by incorporating polarization-dependent parameters into the dual-mode coupling equations. In the nonlinear regime, this linear coupling discrepancy extends to drive the polarization selectivity of THCD: LCP, with higher linear coupling efficiency, exhibits a lower nonlinear excitation threshold and a stronger THCD signal compared to RCP. This direct correlation between linear coupling behavior and nonlinear THCD response not only rationalizes the CMT-based modeling of LCP-RCP coupling but also reinforces the physical basis of THCD as a core claim of this work. The coupling between LCP and RCP components in the nonlinear regime is explicitly modeled by decomposing the nonlinear source term into circular polarization bases. Due to the $C_{4}$ symmetry and structural handedness, the fundamental LCP mode primarily drives a specific nonlinear polarization component that radiates into the 3ω RCP channel. This cross-polarization coupling is the physical origin of the THCD demonstrated in our metasurface. As described in Equation (5), the coefficients $\kappa_{R(L)}\left(3\omega \right)$ depend on the microscopic symmetry of nonlinearities and the symmetry of the meta-atom and lattice. Asymmetric structural design can maximize the chiral contrast by enhancing the right-handed nonlinear response $|N_{R}^{\left(3\omega \right)}{|}^{2}{\left|M_{R}\right|}^{6}$ and suppressing the left-handed contribution $|N_{L}^{\left(3\omega \right)}{|}^{2}{\left|M_{L}\right|}^{6}$, as defined in the chiral contrast formula. This approach ensures the numerator of $CD^{\left(3\omega \right)}$ is dominated by the right-handed mode, while the denominator is also primarily influenced by the right-handed mode, thereby maximizing the overall chirality.

## 3. Results and Discussion

### 3.1. Energy Band and Q-Factor

**Figure 2:**
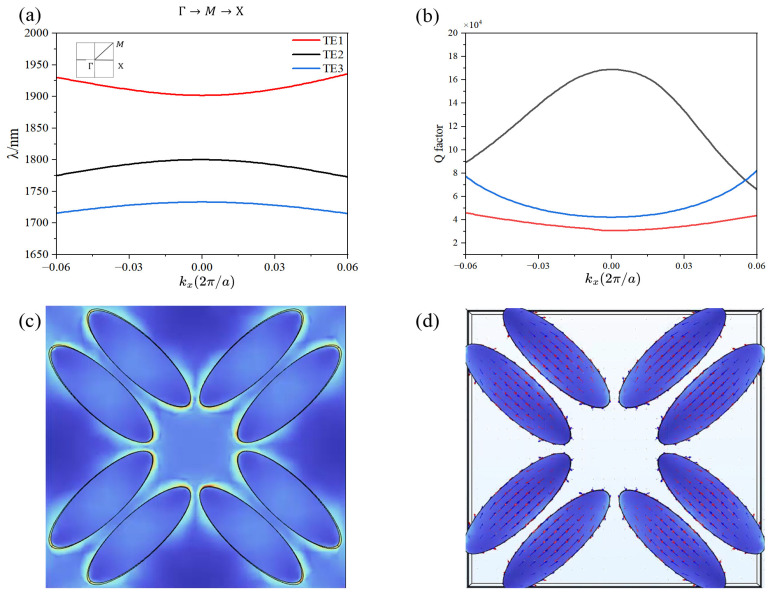
(**a**) Photonic band structure of the eigenmodes under normal incidence ($k_{z}=0$), calculated using FEM. (**b**) Quality factor of the symmetry-protected BIC as a function of normalized height deviation δH, showing asymmetric behavior due to non-symmetric radiation loss. (**c**) Electric-field amplitude distribution of the BIC mode at λ=1800nm, exhibiting strong field confinement. (**d**) Real (red) and imaginary (blue) components of the polarization vector, confirming the dark-mode nature of the BIC.

We conducted a detailed analysis of the structural band characteristics and the associated quality factors of the proposed metasurface. The metasurface, designed with elliptical Si nanorods arranged in a $C_{4}$ symmetric configuration, supports bound states in the continuum that significantly enhance the nonlinear chiral response. Figure 2a is generated via a FEM-based eigenfrequency solver for structural band simulation. We built a 3D FEM model of the $C_{4}$-symmetric Si nanorod unit cell, applied periodic boundary conditions (*x/y* directions) and PMLs (*z* direction), and solved for eigenmodes across the $\left(\Gamma \to M\to X\right)$ Brillouin zone path [51]. This maps eigenfrequencies (wavelengths) to wave vectors, revealing the symmetry-protected BIC. Back conversion from 3ω to ω is neglected due to Si’s weak $\chi^{\left(3\right)}$ ($2.45\times 10^{-19}$ m^2^/V^2^) and low incident intensity ($5\;{\mathrm{kW}/\mathrm{cm}}^{2}$), making it negligible to the modal amplitude and thus having a negligible impact on the main results.

Figure 2a presents the photonic band structure of the metasurface, revealing a symmetry-protected BIC at λ≈1800 nm with a numerically extracted *Q*-factor of $1.8\times 10^{5}$. This ultrahigh *Q* originates from the vanishing coupling between the bound eigenmode and the free-space continuum, yielding an extremely narrow linewidth and prolonged photon lifetime which leads to efficient third-order nonlinearity. The Q-factor curve exhibits asymmetry with respect to height deviation δH due to non-symmetric radiation loss. δH stands for the relative height difference between adjacent rods, defined as $\delta H=\left(H_{2}-H_{1}\right)/H_{1}$, where $H_{2}$ represents the height of the second rod, and $H_{1}$ represents the height of the first rod, which is fixed at 290 nm. The range δH = −0.1 to 0.1 is dimensionless, corresponding to height variation of $H_{2}$ from 261 nm to 319 nm. Due to the existence of substrate, Figure 2a is not symmetric. When δH > 0, the increased overlap between the electromagnetic field and the substrate enhances radiative loss, leading to a sharp drop in Q. Conversely, when δH < 0, the field–substrate interaction weakens, resulting in a more gradual Q-decline. Figure 2c,d display the corresponding electric-field profile. The in-plane snapshot shows pronounced field hot-spots localized at the two apexes of each elliptical rod, consistent with an electric-dipole resonance that is effectively decoupled from radiation.

To confirm the chiral nature of this dark mode, we extract the complex polarization vector P=Re(P)+iIm(P) inside the rod. Figure 2d illustrates that Re(P) and Im(P) exhibit an anti-parallel spatial distribution—the hallmark of a BIC “dark mode” whose radiation is suppressed by destructive interference.

### 3.2. Linear and Nonlinear Characteristics

**Figure 3:**
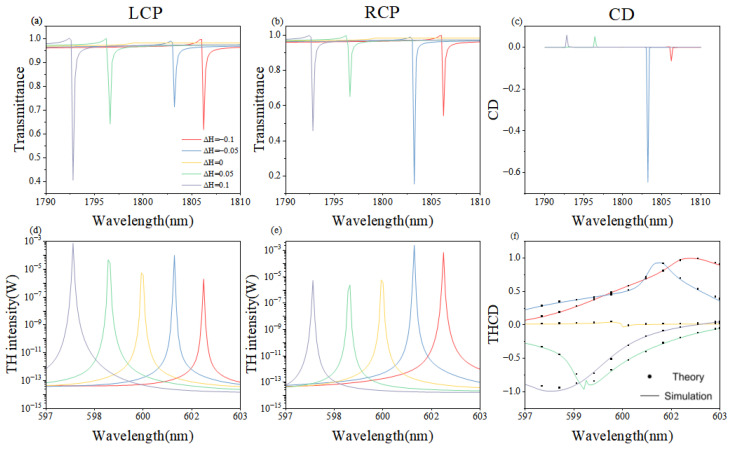
(**a**–**f**) Simulated linear and nonlinear responses as a function of nanorod height deviation δH, defined as $\delta H=\left(H_{2}-H_{1}\right)/H_{1}$, where $H_{2}$ and $H_{1}$ are the heights of the second and first nanorods, respectively, with δH varying from −0.1 to 0.1. (**a**,**b**) Transmittance at the fundamental frequency under RCP and LCP incidence, respectively. (**d**,**e**) THG intensity under RCP and LCP incidence. (**c**) Linear circular dichroism (CD) at the fundamental frequency. (**f**) Third-harmonic CD (THG CD) comparing simulation (FEM) and theory (TCMT).

Figure 3 presents the calculated fundamental-frequency and third-harmonic transmission together with the associated circular-dichroism (CD) spectra as the nanorod height is continuously detuned from −0.1 to 0.1. At the symmetric configuration (δH=0), the fundamental transmission peaks vanish, providing unambiguous evidence for a symmetry-protected bound state in the continuum. Upon departure from this point, the fundamental resonance shifts linearly with height, while the linear CD, defined as $\mathrm{CD}=\left(T_{L}-T_{R}\right)/\left(T_{L}+T_{R}\right)$, attains a maximum magnitude of −0.65 at δH=−0.05 ($H_{2}=275.5$ nm). In the third-harmonic band, the nonlinear emission exhibits a non-uniform variation; nevertheless, the nonlinear CD, which is defined as $\mathrm{THCD}=\left(P_{R}^{\mathrm{TH}}-P_{L}^{\mathrm{TH}}\right)/\left(P_{R}^{\mathrm{TH}}+P_{L}^{\mathrm{TH}}\right)$, is strongly enhanced and approaches unity, thereby demonstrating the concurrent realization of optimal chirality and efficient frequency conversion within a single, highly symmetric metasurface.

Regarding the third-harmonic emission, we have verified that the metasurface supports a supporting quasi-mode at the frequency 3ω. Although this mode possesses a lower Q-factor than the fundamental BIC mode, its existence is crucial, as it facilitates the radiation of the nonlinear polarization $P^{\left(3\omega \right)}$ into the far-field. This mode characterizes the third-harmonic vector $N^{\left(3\omega \right)}$, thereby validating the use of the TCMT framework for evaluating nonlinear power conversion.

It is worth noting that the non-uniform variations in nonlinear intensity and CD enhancement are by no means accidental. Detailed analysis of the electromagnetic-field distribution reveals that the coupling between the metasurface unit cells and their surroundings oscillates periodically with height. At δH=−0.05, the localized surface-plasmon resonance resonantly couples to the BIC modes, giving rise to the pronounced maximum of the fundamental-band CD.

**Figure 4:**
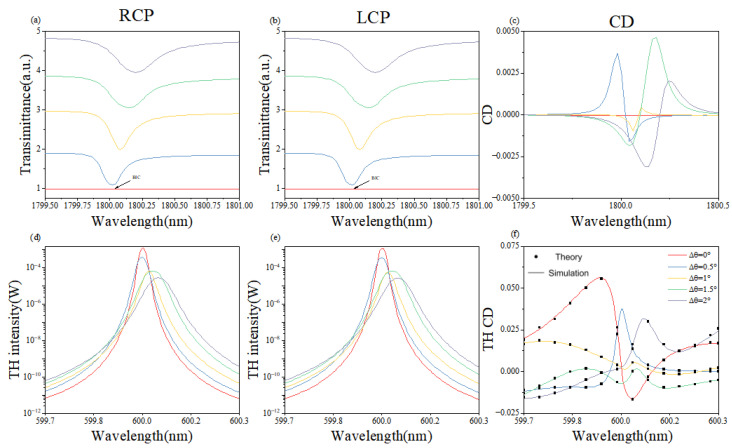
(**a**–**f**) Simulated linear and nonlinear responses as a function of in-plane rotation angle δθ, defined as the angle between each nanorod and the y-axis, varied from 0° to 2°. (**a**,**b**) Transmittance at the fundamental frequency under RCP and LCP incidence. (**d**,**e**) THG intensity under RCP and LCP incidence. (**c**) Linear CD at the fundamental frequency. (**f**) THG CD comparing simulation (FEM) and theory (TCMT). THG is observed even at $\theta =0^{\circ }$ due to nonlinear polarization enhanced by the ultrahigh Q-factor of the dark BIC mode.

Figure 4 summarizes the simulated fundamental-frequency and third-harmonic transmission spectra and CD as the in-plane rotation angle θ is tuned from $0^{\circ }$ to $2^{\circ }$. Across this range, the fundamental-band transmission peaks translate uniformly. At $\delta \theta =0^{\circ }$, the peaks vanish, confirming the symmetry-protected BIC. However, the substrate-induced symmetry breaking leads to the existence of THG, even at θ = 0°. In contrast to height tuning, rotation yields only a modest fundamental-band CD of 0.005 at $\delta \theta =1.5^{\circ }$. In the third-harmonic band, the nonlinear intensity evolves non-uniformly; nevertheless, the nonlinear CD is enhanced several-fold, as illustrated in Figure 4f.

In Figure 4, the observation of maximum THG intensity at $\theta =0^{\circ }$ is a key physical highlight. While the symmetry-protected BIC is a ‘dark state’ that cannot be excited by a linear external field at normal incidence, any infinitesimal perturbation or the symmetry of the nonlinear polarization $P^{\left(3\omega \right)}$ can break the linear selection rules. At this BIC point, the ultra-high Q-factor induces extreme energy localization within the nanostructures. This localized energy drives an intense nonlinear polarization source, which effectively radiates as a third-harmonic signal despite the mode’s ‘dark’ nature in the linear domain.

Figure 3 and Figure 4 together reveal why rotation produces a weak fundamental-band circular dichroism. Angular tuning primarily perturbs the symmetry breaking of the metasurface, whereas height tuning directly reshapes the electromagnetic resonances; the former therefore couples only indirectly to the chiral fundamental mode. In the third-harmonic band, however, the anisotropy introduced by rotation improves the phase-matching landscape and yields a pronounced enhancement of the nonlinear CD.

Consequently, height adjustment amplifies the chiral response within a narrow spectral window, while angle modulation offers a versatile handle when the fundamental CD is intrinsically small. Combining both degrees of freedom enables simultaneous achievement of near-unity chirality and high structural nonlinear conversion.

**Figure 5:**
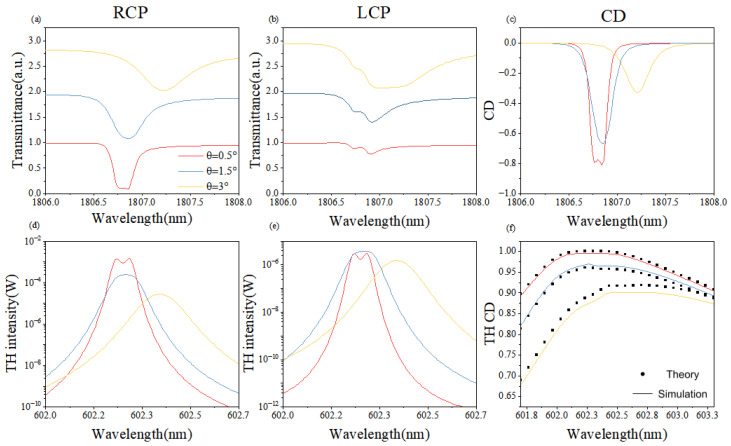
(**a**–**f**) Simulated responses under combined height and angle tuning, with δH=−0.1 fixed and δθ varied from 0.5° to 3°. (**a**,**b**) Transmittance at the fundamental frequency under RCP and LCP incidence. (**d**,**e**) THG intensity under RCP and LCP incidence. (**c**) Linear CD at the fundamental frequency. (**f**) THG CD comparing simulation (FEM) and theory (TCMT). A bimodal resonance appears due to critical coupling between the BIC and radiation modes. THG CD is amplified from −0.8 to ∼1 at $\theta =0.5^{\circ }$.

Figure 5 presents the simulated fundamental-frequency and third-harmonic transmission spectra and CD for a metasurface with δH=−0.1 fixed while the rotation angle is swept. A bimodal resonance appears in the transmission (Figure 5a,d), which we attribute to critical coupling between the metasurface mode and the quasi-BIC mode when their radiative and intrinsic losses are matched. At $\delta \theta =0.5^{\circ }$, the nonlinear CD is amplified from −0.8 to ∼1, and at $\delta \theta =1.5^{\circ }$, it rises from −0.7 to 0.95, confirming the efficient amplification mechanism. Concurrently, the normalized nonlinear conversion efficiency reaches $7\times 10^{-5}$ (Figure 5e).

The remarkable agreement between the FEM simulations and TCMT analytical results in Figure 3f, Figure 4f and Figure 5f is achieved without any adjustable calibration parameters. All required TCMT quantities were pre-determined by fitting the linear scatterings spectra: $\omega_{0}$ and $\gamma_{tot}$ were identified from the Fano resonance peak and linewidth, while $\gamma_{rad}$ was isolated by evaluating the symmetry-breaking degree δθ. These independently extracted values were then used to calculate the THG efficiency, ensuring that the theoretical model remains a rigorous reflection of the physical system’s underlying mechanism. The slight discrepancy observed at $\theta =3^{\circ }$ is attributed to finite mesh resolution and approximate dispersion-neglected $\chi^{\left(3\right)}$ values; upcoming refinements will employ an adaptive grid and dispersion-extracted nonlinear coefficients measured by spectroscopic ellipsometry. By integrating simulation with theory, we systematically tuned the coupling strengths between the incident waves and the metasurface modes, thereby achieving pronounced amplification of both nonlinear chirality and harmonic signals.

### 3.3. Enhancement Effect Analysis

To quantitatively assess the nonlinear performance, all THG conversion efficiencies in this study are evaluated using a normalized protocol. By setting the incident pump power to a standard 1 W in the simulations, the resulting nonlinear output power directly serves as the intrinsic conversion efficiency of the structure. This normalization approach acts as a rigorous enhancement factor, as it isolates the structural contribution to the nonlinear response from the magnitude of external excitation.

Under this standardized framework, the metasurface achieves an optimized efficiency of $7\times 10^{-5}$ at a remarkably low pump intensity of $5\;\mathrm{kW}/{\mathrm{cm}}^{2}$. This value represents a significant enhancement compared to non-resonant chiral designs, which typically exhibit efficiencies below $1\times 10^{-6}$ under similar conditions. The fundamental origin of this boosting effect is the critical coupling condition achieved via precise structural tuning, which perfectly matches radiative and intrinsic losses to maximize electromagnetic energy confinement within the silicon nanorods.

The enhancement of chirality and THG is primarily regulated by geometric variables, such as the height and rotation angle of the nanorods, which respectively control the radiative loss and phase-matching conditions of the quasi-BIC mode. These variables can be precisely tuned using high-precision electron-beam lithography to achieve optimized performance. Additionally, external excitation conditions, including pump intensity and wavelength alignment, serve as active control methods to modulate the nonlinear output.

To evaluate the competitive edge of our $C_{4}$-symmetric metasurface, we provide a comprehensive comparison with other state-of-the-art BIC-enhanced nonlinear devices in Table 1. It is noteworthy that previous works, such as that by Liu et al. [47], achieve impressive conversion efficiencies but typically require intense pump radiation in the ${\mathrm{MW}/\mathrm{cm}}^{2}$ or even ${\mathrm{GW}/\mathrm{cm}}^{2}$ regime. In stark contrast, our structure maintains a high nonlinear conversion efficiency of $7\times 10^{-5}$ at an exceptionally low pump intensity of only $5\;{\mathrm{kW}/\mathrm{cm}}^{2}$. This “low-power flexibility” is a direct result of the ultra-high Q-factor and the optimized critical coupling achieved through our symmetry-breaking strategy. Such performance underscores the potential of our design for integration into power-sensitive nanophotonic circuits where minimizing thermal effects and energy consumption is paramount.

To further advance this research, our ongoing work focuses on the experimental validation of the proposed $C_{4}$-symmetric metasurface. Preliminary progress has been made in fabricating the silicon nanorod arrays, with a focus on evaluating fabrication tolerances [52]. Future research will explore the integration of these chiral metasurfaces with silicon-on-insulator (SOI) waveguides to develop miniaturized, chip-scale nonlinear photonic devices with integrated quantum information processing.

**Table 1:** Comparison of different works on THCD and nonlinear conversion efficiency.

BIC Type	THG CD	Pump Intensity	Nonlinear Conversion Efficiency	Reference
Accidental	∼0.99	$1\;\mathrm{MW}/{\mathrm{cm}}^{2}$	∼1.1 $\times \;10^{-2}$	[53]
Symmetry-protected	∼0.948	$1.8\;\mathrm{MW}/{\mathrm{cm}}^{2}$	∼2.99 $\times \;10^{-2}$	[54]
Symmetry-protected	Not reported	$10\;\mathrm{kW}/{\mathrm{cm}}^{2}$	∼6 $\times \;10^{-5}$	[45]
Symmetry-protected	∼0.97	Not reported	Not reported	[48]
Symmetry-protected	∼0.9	$1\;\mathrm{MW}/{\mathrm{cm}}^{2}$	∼4.5 $\times \;10^{-3}$	[55]
Symmetry-protected	>0.8	Not reported	∼1 $\times \;10^{-2}$	[56]
Symmetry-protected	∼0.99	$5\;\mathrm{kW}/{\mathrm{cm}}^{2}$	∼7 $\times \;10^{-5}$	Our work

## 4. Conclusions

In summary, we have demonstrated a $C_{4}$-symmetric chiral metasurface that leverages symmetry-protected quasi-BIC resonances to overcome the inherent trade-off between optical chirality and resonance *Q*-factors. Our investigation reveals that the observed near-unity circular dichroism (0.99) and the significantly boosted THG conversion efficiency ($7\times 10^{-5}$ under a low pump intensity of 5 kW/cm^2^) are rooted in the critical coupling condition achieved via precise structural tuning. By systematically adjusting the geometric parameters of the nanorod array, we successfully matched the radiative loss of the quasi-BIC mode with its intrinsic loss, leading to maximum electromagnetic energy confinement within the silicon resonators. This loss-matching mechanism not only maximizes the chiral light–matter interaction but also provides the requisite local field enhancement for efficient nonlinear conversion. These findings provide a robust theoretical framework for manipulating light–matter interactions at the nanoscale and offer a practical route for developing ultra-compact, high-performance nonlinear chiral photonic devices for future integrated quantum optical systems.

## Data Availability

The data underlying the results presented in this paper are not publicly available at this time but may be obtained from the authors upon reasonable request.

## Abbreviations

The following abbreviations are used in this manuscript:

BIC	Bound states in the continuum
CD	Circular dichroism
RCP	Right-circularly polarized
LCP	Left-circularly polarized
TCMT	Time-domain coupled-mode theory
SHG	Second harmonic generation
THG	Third harmonic generation
FF	Fundamental frequency
